# Evidence for the role of sound on the growth and signal response in duckweed

**DOI:** 10.1080/15592324.2022.2163346

**Published:** 2023-01-12

**Authors:** Zi Ye, Rui Yang, Ying Xue, Ziyi Xu, Yuman He, Xinglin Chen, Qiuting Ren, Jinge Sun, Xu Ma, Jerri Hu, Lin Yang

**Affiliations:** a College of Music, Film & Television, Tianjin Normal University, Tianjin, China; b Tianjin Key Laboratory of Animal and Plant Resistance, College of Life Sciences, Tianjin Normal University, Tianjin, China; c Tianjin Radiant Banyan Development Centre for Children with Special Needs, Tianjin, China

**Keywords:** Sound, duckweed, signal, glu, Ca2+

## Abstract

Sound vibration, an external mechanical force, has been proven to modulate plant growth and development like rain, wind, and vibration. However, the role of sound on plants, especially on signal response, has been usually neglected in research. Herein, we investigated the growth state, gene expression, and signal response in duckweed treated with soft music. The protein content in duckweed after music treatment for 7 days was about 1.6 times that in duckweed without music treatment. Additionally, the potential maximum photochemical efficiency of photosystem II (Fv/Fm) ratio in duckweed treated with music was 0.78, which was significantly higher in comparison with the control group (P < .01). Interestingly, music promoted the Glu and Ca signaling response. To further explore the global molecular mechanism, we performed transcriptome analysis and the library preparations were sequenced on an Illumina Hiseq platform. A total of 1296 differentially expressed genes (DEGs) were found for all these investigated genes in duckweed treated with music compared to the control group. Among these, up-regulation of the expression of metabolism-related genes related to glycolysis, cell wall biosynthesis, oxidative phosphorylation, and pentose phosphate pathways were found. Overall, these results provided a molecular basis to music-triggered signal response, transcriptomic, and growth changes in duckweed, which also highlighted the potential of music as an environmentally friendly stimulus to promote improved protein production in duckweed.

## Introduction

Sound is an oscillatory concussive pressure wave transmitted through gas, liquid, and solid. The influence of environmental factors such as water, wind, light, and predation on plant growth has been well studied^1^. Plants could respond to stimuli, such as wind, light, and sound.^2^ It has been shown that plants can perceive external sounds and some can even make sounds through the xylem^3^ and communicate information through sound.^4^ Sound transmitted by liquid, gas, and solid. Previous studies showed the effects of sound in animals and plants. Sound affected the growth and physiological development of plants. The cabbage and cucumber at the seedling and maturity stages were treated with sound, and it was found that oxygen uptake by the plants was significantly increased at both stages.^5^ Different types of sound enhanced plant survival,^6^ directed plant root growth,^7^ and shorted plant germination,^8^ and thus sound influenced plant development. It has also been shown that certain sounds can affect fruit development. There were several examples including delaying fruit ripening,^7,9^ increasing the nutrient content of the fruit,^10^ and affecting flower pollination.^11^ However, further investigation addressing the mechanism of music influence on plant growth needs to be addressed.

It has been reported that sound also caused changes in the biochemical and genetic levels of plants. Sound has played a role in altering plant cell cycle, stomatal opening, growth hormone release, enzyme and hormone activity, immune function, RNA content, and transcript levels:^1,12,13^ (1) Sound altered the cell cycle of plants, accelerating protoplasmic movement within plant leaves^14^ and speeding up plant metabolism. (2) Sound affected the stomatal opening of plant leaves,^12^ which led to faster uptake of nutrients and water.^15^ It has also allowed plants to absorb herbicides and pesticides, leading to the effective use of herbicides and pesticides. (3) The data suggested that some responses elicited by sound vibrations were modulated by specific changes in phytohormone levels, which also caused changes in phytohormone signaling like some stimuli.^13^ For example, the concentration of the salicylic acid increased in *Arabidopsis* under the influence of the sound of 1000 Hertz with 100 decibels, which increased plant defense and enhanced plant disease resistance.^16^ (4) In response to sound stimulation, some genes were activated at the transcriptional level,^17^ resulting in the changes in genes involved in the cellular metabolism of plants. Changes in gene expression and proteomics of *Arabidopsis thaliana* exposed to acoustic waves were studied. The expression of enzymes related to scavenging reactive oxygen species (ROS) and primary metabolism (tricarboxylic acid cycle, ATP synthesis, amino acid metabolism, and photosynthesis) has been upregulated.^13^ This showed that sound had great potential for application in the field of plant biology. Therefore, it is important to figure out what molecular signaling processes activate those genes in plant growth during sound such as music environment.

Unlike animals with a rapid nervous system that responds to the environment, plants process several molecular signal transduction responses. However, how to respond to sound has not been studied. Glutamate (Glu), an excitatory neurotransmitter in the mammalian central system, has been found to respond to the environment and play a role during plant growth. Glu has been proved to be a signaling molecule in plant cells and played an important role in seed germination and cellular metabolism.^18,19^ Glu responds to mechanical damage, cold stress, and heat stress.^19–21^ An interesting signal response caused by Glu was that it led to Calcium (Ca) signal responses throughout a whole plant body after wounding.^20^ This was due to Ca^2+^ flux depending on glutamate receptor-like proteins (GLR), Ca^2+^ permeable channels. Ca is one of the most versatile signals in living organisms,^22^ which play a significant role in the growth and development of plants in response to the environment: (1) Ca^2+^ was a key regulator, and its concentration in eukaryotic cells was significantly regulated by the Ca^2+^ sensor by the hormone;^22,23^ (2) Ca^2+^ binding protein signaling pathways mitigated biotic stresses;^24^ (3) Several tolerance strategies coordinated by Ca^2+^ signal has been found. For example, it responded to salt stress mediated by an enhanced antioxidant.^25^ These facts indicated that intracellular Ca^2+^ played an important role in eukaryotic signaling networks. However, it remains unknown that the response mechanisms of endogenous Glu and Ca^2+^ in duckweed under music treatment. The molecular signal responses in plants during music environment need to be discovered.

Duckweed is a fast-growing aquatic plant, with high environmental adaptability and wide distribution.^26^ Compared to algae, duckweed is more readily available in large quantities, thus saving time and reducing energy consumption.^27^ The advantages of duckweed have high nutrient uptake and are commonly used for wastewater treatment to reduce environmental pollution.^28^ Furthermore, with its high protein and starch accumulation capacity, duckweed is widely used as an ideal raw material for animal feed and ethanol production.^29^ The potential of duckweed as an environmental indicator has also been studied and developed.^30^ However, due to the high protein content of duckweed, which is often used as the feed source, therefore it is especially significant to enhance the content of protein.^31^

In this study, we used *Lemna turionifera 5511* as the material to study the change of duckweed treated with music. The main objectives were as follows: (i) to investigate the phenotypic and the content of protein in duckweed treated with sound treatment; (ii) to examine situations of Ca^2+^ signaling and Glu responses in duckweed treated with music; (iii) to reveal changes of significant metabolic pathways under music treatment; (iv) to study the gene expression changes and response mechanisms in duckweed under music treatment. The results of this study provided insight into how duckweed responded to music and how music can promote growth and the molecular mechanism of duckweed.

## Result

### Enhanced growth of duckweed by sound

**Figure 1.**
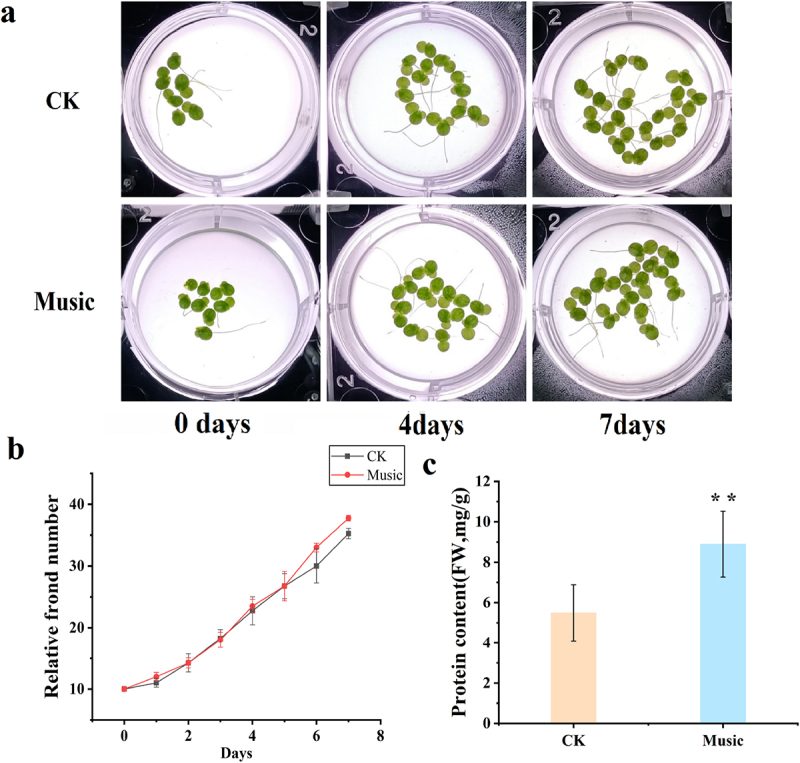
(a) The phenotype of duckweed treated with or without music for 0–7 days; (b) Frond growth curve over 7 days of cultivation treated with or without music; (c) the contents of protein in duckweed treated with or without music were determined by using Coomassie Brilliant Blue staining. The error bars in the graph are standard errors (SE) with biological repetitions n = 5. *P < .05, **P < .01 represent significant differences and extremely significant differences, respectively.

In order to investigate the physiological effects of duckweed treated with music, duckweed treated with or without music in 7 days has been studied. Shown as Figure 1a and b, the frond number of duckweed distinctly increased after the 5th day of sound treatment, although no significant change in phenotype was observed during the first 5 days of music treatment. The fround number of duckweed with music treatment for 6 d was 38, which is 3 more than that treated without music. Duckweed is a potential high-protein feed resource due to the protein content, thus the protein content of duckweeds with or without sound treatment was measured. Shown as Figure 1c, the protein content in duckweeds treated with music after 7 days was significantly higher than the protein content in control duckweeds, which is 1.6 fold of that. These results showed that music in this study has positive effects on the growth of duckweed.

### Enhanced Fv/Fm and the expression of photosynthesis related genes in duckweed under music treatment

**Figure 2.**
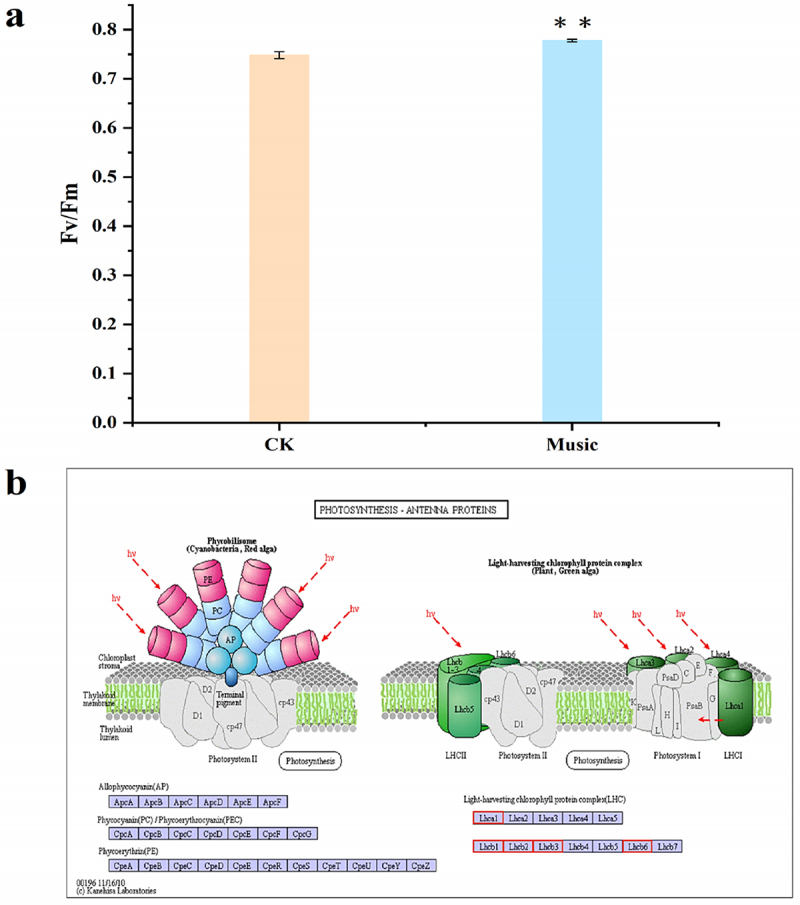
(a) Fv/Fm was determined after treatment with and without music. The error bars in the graph are standard errors (SE) with biological repetitions n = 4. *P < .05, **P < .01 represent significant differences and extremely significant differences; (b) Kyoto Encyclopedia of Genes and Genomes (KEGG) maps the pathways associated with photosynthetic antenna proteins. The box color was determined by the expression pattern of unigenes coding corresponding proteins. Red represents up-regulation, green represents down-regulation, and yellow represents mixed-regulation.

The potential maximum photochemical efficiency of photosystem II (Fv/Fm) has been investigated in this study. As shown in Figure 2a, Fv/Fm test in duckweed treated with music was 0.78, which was significantly higher in comparison with the control group (P < .01).

Figure 2b shows the pathway associated with photosynthesis-antenna proteins. The gene expression difference in photosynthesis-antenna protein-related gene expression has been investigated in the music group compared to that in the control group (CK). Light-harvesting complex II (LHCII) refers to the largest photosynthetic pigment-protein complex of plant photosystem II, which has been up-regulated in the music group. The red box represents up-regulated gene expression. Therefore, these results provided reasonable evidence for elevated photosynthesis during music treatment.

### Glu response and Glu contents under music treatment

**Figure 3.**
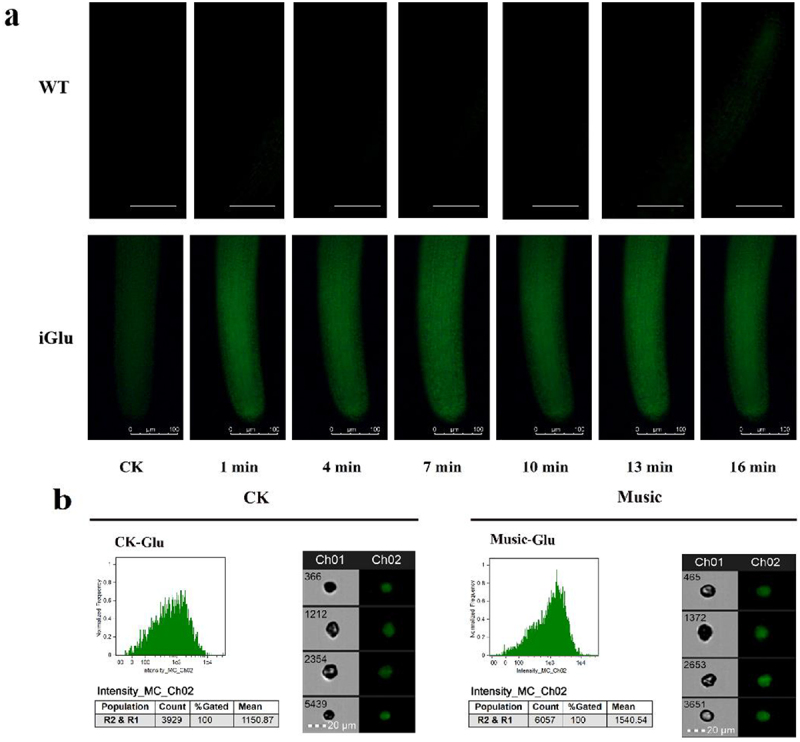
(a) The fluorescence in the iGluSnFR duckweed subjected to transient music for 16 min; (b) the Glu fluorescence intensity of the protoplasts in the roots of iGluSnFR duckweed was detected by flow cytometry, 6 thousand cells each group were analyzed. The iGluSnFR duckweed was treated with or without music for 10 h. The excitation wavelength was 488 nm. Scale bar = 20 μm (Ch 01 is the protoplast in bright, Ch 02 is fluorescently excited tong at 488 nm).

Glu, playing a key role in protein composition, metabolism, and signaling has been investigated during music treatment. GFP-based Glu sensor iGluSnFR transgenic duckweed (iGlu duckweed) was successfully obtained in our previous study.^32^ The fluorescence images of Glu in the roots of iGluSnFR duckweed subjected to transient music for 0–16 min are shown in Figure 3a. The fluorescence intensity changed with music shock. The results indicated that music promoted the Glu signaling response of iGluSnFR duckweed.

To further study the Glu signal response duckweed, the fluorescence intensity of protoplasts extracted from the iGluSnFR duckweed was analyzed under 488 nm excitation(Figure 3b). The fluorescence intensity of iGluSnFR duckweed treated with or without music was 1540.54 and 1150.87, respectively. Thus, quantitative analysis showed that the fluorescence intensity of iGluSnFR duckweed subjected to soothing music was significantly stronger than those treated without music. The result revealed the involvement of the Glu signal during music processing.

### Differentially expressed genes (DEGs) and Gene ontology and KEGG pathway analyses of DEGs

**Figure 4.**
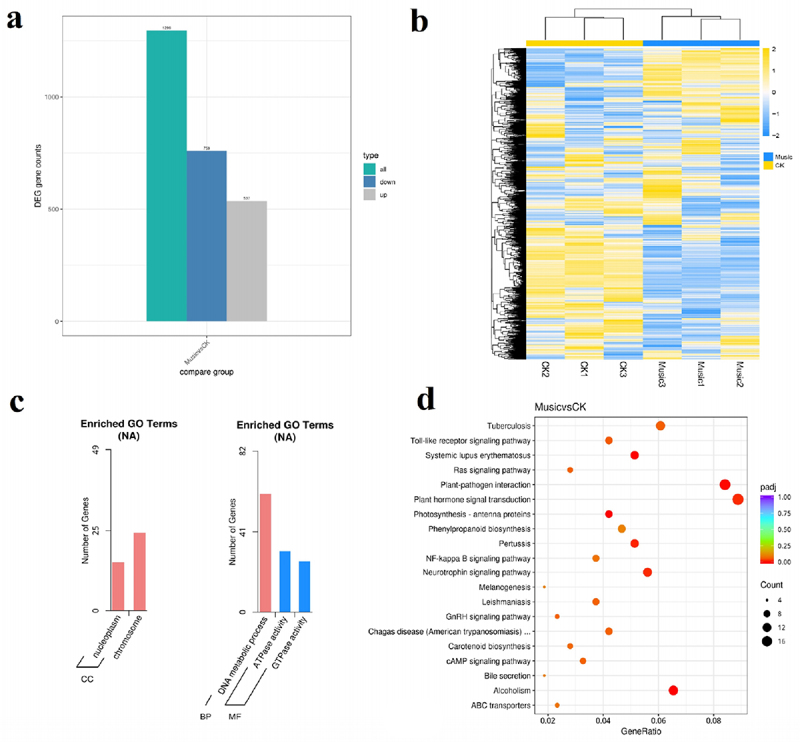
(a) The histogram showed differentially expressed genes in response to music treatment; (b) cluster analysis of DGEs in the “Music” and “CK”; (c) in the case of “Music vs CK”, the number of enriched up-regulated DEGs and down-regulated DEGs in different gene ontology categories. (d) KEGG enrichment scatter plot (Rich factor referred to the ratio of the number of differentially expressed genes in the pathway to the total number of all annotated genes in the pathway).

The DEGs were analyzed and organized to better understand the molecular mechanisms of duckweed under music treatment. As shown in Figure 4a, a total of 1296 DEGs were found in “Music vs CK”, of which 759 were down-regulated genes and 537 were up-regulated genes. In addition, the overall expression profile of the DEGs is observed in Figure 4b, the heat map showed the gene expression levels that were significantly up-regulated and down-regulated in the case of “Music” and “CK”. The darker the red, the higher the expression. The darker the green, the lower the expression.

To further understand the differences of DEGs in duckweed treated with music, GO enrichment analysis was performed on duckweed under music treatment and control group. As shown in Figure 4c, “chromosome” was in the category of the cellular compartment with the most up-regulated DEGs. It was followed by “nucleoplasm” in the category of the cellular compartment. In contrast, the “DNA metabolic process” was in the category of the biological process with the most down-regulated DEGs. It was followed by “ATPase activity”, and “GTPase activity” in the category of molecular function.

Transcriptome analysis was performed to investigate the potential functions of KEGGs and DEGs in duckweed treated with music. The genes involved in plant hormone signal transduction and plant–pathogen interaction were significantly up-regulated and down-regulated, respectively. Among the 20 pathways with the most significant enrichment (Figure 4d), the plant hormone signal transduction and plant–pathogen interaction contained the most differential genes.

### The Ca^2+^ signal response under music treatment

**Figure 5.**
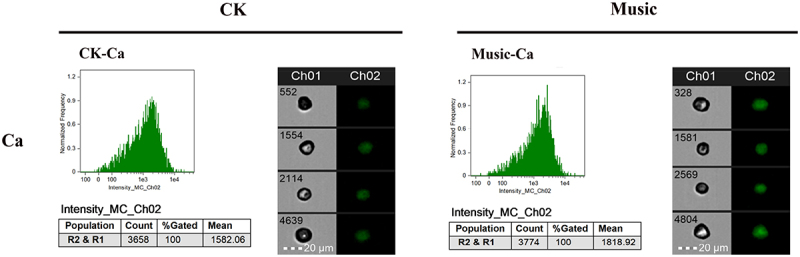
The duckweed was treated with or without music for 10 h and stained by Flou-4 AM. Ca content in protoplast analyzed by flowsight system in 488 nm. Scale bar = 20 μm (Ch 01 is the protoplast in bright, Ch 02 is fluorescently excited tong at 488 nm).

To further investigate the Ca signal response duckweed, the fluorescence intensity of protoplasts extracted from WT duckweed was analyzed under 488 nm excitation (Figure 5). The average fluorescence intensity of 6000 protoplasts in WT duckweed treated with or without music was 1818.92 and 1582.06, respectively. Thus, quantitative analysis showed that the fluorescence intensity of WT duckweed subjected to soothing music was significantly stronger than those treated without music. The result revealed the involvement of the Ca signal during music processing.

**Figure 6.**
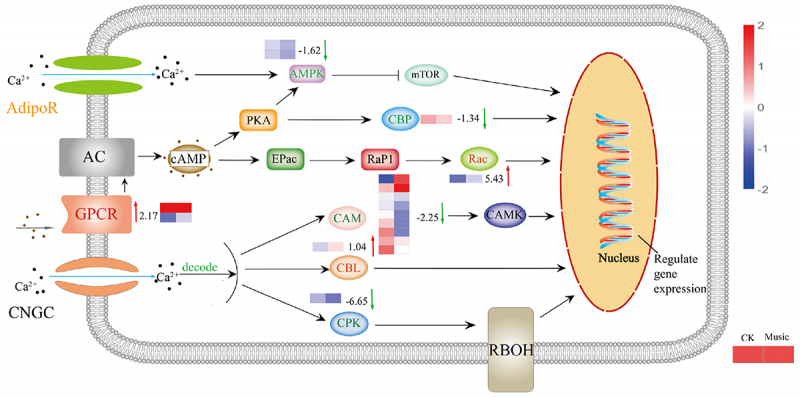
The response of the Ca signal pathway under music treatment. The channels and proteins associated with Ca signal transduction were shown in the figure. The color of letters and arrows represented the change in the DEGs: red indicated up-regulated, green indicated down-regulated, and yellow indicated mix-regulated. The heat map next to the arrow showed the expression of different genes encoded the protein in Music and CK duckweed. Red meant high expression; blue meant low expression. (Rac: Ras-related C3 botulinum toxin substrate 1, AdipoR: adiponectin receptor, CPK: creatine kinase, AMPK: AMP-activated protein kinase, GPCR: GTP-binding protein, CBP: CREB-binding protein, CBL: calcineurin B-like protein, CAM: calmodulin).

To explore the differences in molecular mechanisms between duckweed treated with music and the control group, genes related to the Ca^2+^ signaling pathway were analyzed. As shown in Figure 6, g protein-coupled receptors (GPCRs) were up-regulated, which has raised 2.17 log2 Fold Change, while adiponectin receptor (AdipoR) located on the plasma membrane was mix-regulated. Moreover, calmodulin (CAM) genes, Calcium-dependent protein kinase (CPK) genes, and AMP-activated protein kinase (AMPK) genes were down-regulated. Furthermore, calcineurin B-like protein (CBL) has raised 1.04 log2 Fold Change. Ras-related C3 botulinum toxin substrate 1 (Rac) genes were up-regulated, which has raised 5.43 log2 Fold Change, while CREB-binding protein (CBP) genes were down-regulated. These results suggested that mTOR, CAMK, CBL, Rac, CBP, and RBOH were triggered by intracellular calcium ions, and the expression of proteins related to metabolism has also altered.

### Metabolic pathways of duckweed under music treatment

**Figure 7.**
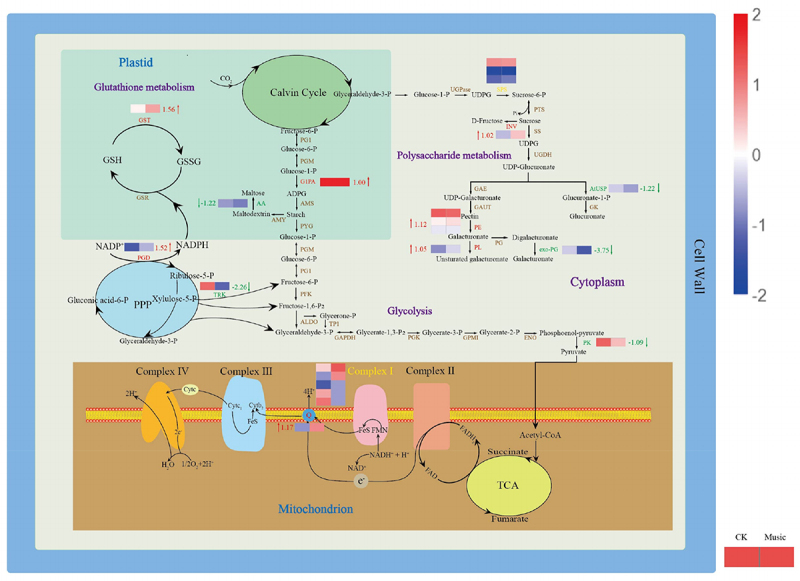
The metabolic mechanism of duckweed was influenced by music. Metabolites (in black) and proteins with enzymes and transporters are shown. The arrows beside or through proteins indicate the directions of catalytic reactions or transportation. Red arrows represent upregulation, green arrows represent downregulation, and yellow arrows represent mixed regulation. The heat map next to the arrow showed the expression of different genes encoded the protein in Music and CK duckweed. Red meant high expression; blue meant low expression. (INV: in+vertase, SPS: sucrose-phosphate synthase, G1PA: glucose-1-phosphate adenylyltransferase, PK: pyruvate kinase, PE: pectinesterase, PL: pectate lyase, exo-PG: galacturan 1,4-alpha-galacturonidase, AtUSP: UDP-sugar pyrophosphorylase, AA: alpha-amylase, GST: glutathione S-transferase, PGD:6-phosphogluconate dehydrogenase, TRK: transketolase, ND5: NADH-ubiquinone oxidoreductase chain 5, NdhB: NAD (p) H-quinone oxidoreductase subunit 2).

Some primary and secondary metabolic pathways related to the molecular mechanism are represented in Figure 7. Starch is considered to be an energy deposit in plants. The gene encoding glucose-1-phosphate adenyltransferase (G1PA) is up-regulated during starch metabolism, which has raised 1.00 log2 Fold Change. However, the gene encoding alpha-amylase (AA) is down-regulated. GSH, with the help of glutathione S-transferase (GST), is transformed into R-S-glutathione. The glutathione S-transferase (GST) gene in glutathione metabolism was up-regulated, which has raised 1.56 log2 Fold Change. Further, the gene encoded 6-phosphogluconate dehydrogenase (PGD) was up-regulated involved in the pentose phosphate pathway, which has raised 1.52 log2 Fold Change. Therefore, it tended to be activated to provide more NADPH under music treatment. The expression of pyruvate kinase (PK) taking part in glycolysis was down-regulated. Additionally, NADH-ubiquinone oxidoreductase chain 5 (ND5) mix-regulated and NAD(P)H-quinone oxidoreductase subunit 2 (NdhB) was up-regulated in the oxidative phosphorylation pathway were also noteworthy, which has raised 1.17 log2 Fold Change. In addition, the biosynthesis of pectin is one of the major branches of sugar metabolic flux, and most of the enzymes in this pathway were up-regulated in duckweed.

## Discussion

The growing price of Soybean meal presents a challenge to researchers to investigate the possibilities for application of green technologies to increase the protein production of plants to replace soybean as the feed resource. The Sound caused changes in the growth and development, biochemical and genetic levels of plants.^1,5^ Researchers found that sound waves significantly affected the number of leaves of mustard greens.^15^ In our preliminary research, we found that different decibels have different effects on the growth of duckweed. Music at too high a decibel level was not beneficial to plants. This was consistent with the study of Wang et al. and Cai et al.^8,33^ In this study, the results showed that the soft music named “The Purple Butterfly” at 60–70 decibels promoted the growth of the frond number of duckweed (Figure 1). Furthermore, under music treatment for 7d, the average protein content in duckweed treated with music was 8.89 mg/g FW, which was significantly higher than the average protein content (5.49 mg/g FW) in duckweed treated without sound treatment (Figure 1c). A similar result has been reported that sound waves enhanced the synthesis of soluble protein in chrysanthemum.^16^ Therefore, music promoted the accumulation of protein in duckweed.

Nucleic acid, the hereditary material, stores a digital record of every protein’s design. The transcript is important for protein expression.^34^In our research, we studied the effects of soft music on transcription. GO enrichment analysis has been performed and it showed that ‘chromosome’ was in the category of the cellular compartment with the most up-regulated DEGs (Figure 4c). Thus, the enhancement of protein accumulation by music might be related to the DEGs associated with chromosome synthesis being up-regulated. The findings demonstrated that music increased the utilization of duckweed in terms of its use as the feed source.

Fv/Fm ratio reflects the maximum light energy conversion efficiency of the PS II reaction center. Meng found that Fv/Fm ratio was increased of strawberry leaves under the sound wave,^35^ which was similar to our research. Fv/Fm ratio in duckweed treated with music was higher than that of CK (Figure 2a), which suggested that music enhanced the activity of the PS II reaction center and the ability to use light energy. In addition, most of the DEGs related to ‘Photosynthesis-antenna proteins’ were up-regulated (Figure 2b). This result suggested that antenna protein genes were closely associated with increased photosynthesis under music. Notably, in a previous study, Lhcb6 has been shown to be involved in reducing oxidative stress and photoprotection under natural conditions.^36^ In addition to this, it has been found that Lhcb2 was associated with a light-harvesting complex playing a critical role in providing the energy required for photolysis.^13^ Therefore, music stimulation promoted photosynthesis by increasing the expression of photosynthesis-related genes with music treatment.

The understanding of the genome of duckweed has greatly facilitated the application of phylogenetic studies.^37^ And genome of duckweed provided a great opportunity to investigate how different levels of metabolism translate into molecular changes of genes.^38^ In the present research, functional annotation of DEGs via the database of KEGG revealed that “Plant hormone signal transduction” was highly enriched in the music group compared with control (Figure 4d). A similar result has been reported that most of the genes involved in hormonal signaling were also up-regulated in *Arabidopsis* treated with sound.^13^ Nevertheless, how music affected the expression of genes related to hormone signaling in duckweed remained to be further studied.

Plants could sense local signals like wounding, and transmit the signal to the whole plant body rapidly by Glu and Ca^2+^ signal.^20^ Our results showed that music stimulated the Glu signaling response in iGluSnFR duckweed (Figure 3a). We investigated changes in the Glu content of duckweed treated with music. The results showed that music enhanced the content of the signaling Glu (Figure 3b). Also, we studied the signal responds to different music in this similar sound with 60–70 decibels (Fig. S2), which also showed that sound stimulated the signal response. Exactly, the Ca^2+^ signal, which could be released by GLR with Glu activation, acts as a second messenger and regulator in most signaling networks. The localization of Ca^2+^ stained by Fluo-4-AM showed that music increased the amount of Ca^2+^ accumulation (Figure 5). Toyota et al. (2018) showed that mechanical stimulation elicited a change in plant Glu response, which led to a change in Ca^2+^ concentration to induce a local plant response. We noticed significant up-regulation of the CBL gene in duckweed, calcineurin B-like protein (CBL), which interacted with CBL-interacting protein kinases (CIPKs) to facilitate downstream signaling. Functions as a key component in the regulation of various stimuli or signals in plants.^39^ Our results suggested that the second messenger Ca^2+^ may be involved in the signaling of musical stimuli. Among them, G protein-coupled receptors (GPCRs) were also significantly up-regulated, affecting the influx of calcium ions and promoting signal transmission. Ghosh et al. (2016) have also shown that activation of ion transport proteins played a role in sound-mediated responses. In addition, calmodulin (CAM) and calcium-dependent protein kinase (CPK) were significantly down-regulated (Figure 6), which played regulatory roles in a variety of cellular processes, affecting plant stress resistance and growth and development.^40,41^ Yamauchi et al. (2017) showed an NADPH Oxidase RBOH Functions in Rice Roots.^42^ However, the mechanisms underlying the regulation of resistance in duckweed under the influence of music still need to be further explored.

This study provided the first comprehensive transcriptome analysis of *Lemna turionifera 5511* exposed to music. Transcriptome analysis identified several biological processes involved in response to musical stimuli. Researchers have found that stimulation by external factors may lead to changes in the proteome associated with primary metabolism in plants.^43^ This was similar to the results of our study. These results showed that genes involved in the basic biological processes of carbohydrate metabolism and protein metabolism respond to musical stimuli, with most of them being up-regulated (Figure 7). For example, the transcript levels of some genes involved in cellular in the pentose phosphate pathway and cell wall biosynthesis were up-regulated. Previous studies have shown that sound increased the content of soluble sugars in chrysanthemums.^17^ In the present experiment, genes involved in starch and sucrose metabolism were up-regulated, also suggesting that music promoted carbohydrate accumulation in plants. ATP, a commonly used carrier of energy in cells, is produced through two main pathways: light reactions in chloroplasts and oxidative phosphorylation in mitochondria. The transcript levels of the genes encoding the enzymes involved in oxidative phosphorylation were up-regulated in this study, suggesting that ATP content may be enhanced in response to musical stimulation during electron transport in the respiratory chain. This may be one of the reasons for the accelerated metabolic response. Our study identified changes in the metabolic pathways of *Lemna turionifera 5511* in response to musical stimulation through transcriptome analysis. The use of music to stimulate the expression of plant genes and promote plant growth and metabolism offers new ideas for the development of ecological agriculture.

In conclusion, we explored frond number, protein content, photosynthetic capacity, Glu and Ca^2+^ signaling response, gene analysis, and metabolic pathway analysis during sound environment. Our data has allowed us to confirm music enhanced growth, especially protein content, by enhancing the expression of photosynthesis-related genes and stimulating the Glu signal response of duckweed. Delving into the fields of plant production and protein enhancement, we gained insight into the function of music. These results provided new ideas for research in the field of plant acoustics.

## Materials and methods

### Duckweed cultivation and sound processing

The duckweeds (*Lemna turionifera 5511*) were cultured in our lab in a sterile environment following Yang et al. (2013). The duckweed was treated with or without the music named “The Purple Butterfly” for 7 days. Music was played for five hours a day, with the sound of 60–70 decibels, and 100–500 Hz. The culture conditions were with a light intensity of 62 μmol m^−2^ s^−1^ and a temperature of 20/28°C. The independent samples of duckweed for at least 3 treatments for each group were measured in the following research for photosynthsis, protein content, Ca and Glu measurement. For the measurement of the photosynthetic system and the observation of iGluSnFR duckweed by fluorescence microscope, 20 groups of duckweed were studied. For determination of protein content, Ca and Glu content measurement, 0.5 g (about 40 groups of duckweed) duckweed for each experiment has been obtained.

### Measurement of the photosynthetic system

Photosynthetic fluorescence parameters were measured in the duckweeds after music treatment. The samples were treated in dark for 30 min to deplete the organic matter in the leaves before measurement. Subsequently, the maximum quantum yield of photosystem II (PSII) of duckweed leaves was measured by Dual-PAM100 fluorometer (Waltz Company, Germany).

### Determination of protein content

The duckweeds (0.5 g) treated with or without music were ground with 5 ml 0.05 M phosphate-buffered saline (PBS) and the protein was extracted. After adding Coomassie Brilliant Blue G-250 staining solution to the standards and samples, they were mixed for 5 min. A series of gradients of standard protein solutions were prepared using standard bovine serum proteins. The absorbance of the standards and samples at 595 nm was recorded using an Enzyme-labeled Instrument. A standard curve was generated by plotting the average absorbance at 595 nm as a function of the concentration of the protein standard. The sample protein content can be calculated from the standard curve.

### Music promotes the Glu response of duckweed

The iGluSnFR duckweed was treated with music 10 h in advance. And then we carried out experimental observations in a musical setting. Fluorescence changes in the roots of the duckweed under the influence of transient music were observed using a fluorescence microscope (Leica, DFC450C, MD5000, Berlin, Germany).

### Flow cytometric analysis of Ca and Glu content in duckweed

Protoplast extraction: duckweed was treated with the music for 10 h. The duckweed was fixed in 95% ethanol for 15 min, and then washed 2–3 times by adding DPBS. The roots and leaves were separated and enzymatically digested by adding 1% pectinase and 1% cellulase at 37°C for 60 min in dark. The obtained protoplasts were washed three times with DPBS and then filtered through a 200-mesh cell sieve.

Glu content in protoplasts of duckweed analysis: The protoplasts of iGluSnFR duckweed treated with or without music were analyzed by FlowSight (Merck millipore, FlowSight® imaging flow cytometer, Germany). The exciting wavelength was 488 nm. Six thousand protoplasts in each group were analyzed and six parallel groups were made. Ca^2+^ content in protoplasts of duckweed analysis: The WT duckweed was stained with Fluo-4-AM dye at 37°C in the dark for 1 h. The protoplasts of WT duckweed treated with or without music were analyzed by FlowSight (Merck millipore, FlowSight® imaging flow cytometer, Germany). The exciting wavelength was 488 nm. Six thousand protoplasts in each group were analyzed and six parallel groups were made.

### RNA library sequencing and quality control

The duckweeds in three independent parallel experiments treated with or without music were frozen in a − 80°C refrigerator and then sequenced at Novogene (Chaoyang, Beijing). Total RNA was extracted using the RNAprep Pure Plant Kit (TIANGEN, Beijing, China). RNA degradation and contamination were monitored on 1% agarose gel. RNA purity was checked using the NanoPhotometer® spectrophotometer (IMPLEN, CA, USA). RNA was sonicated using Bioruptor Pico (Diagenode), for 15 cycles with 30 s on and 30 s off. Sequencing libraries were generated using the NEBNext® Ultra™ RNA Library Prep Kit (NEB, USA). After library construction, RNA integrity was assessed by the RNA Nano 6000 Assay Kit of the Agilent Bioanalyzer 2100 system (Agilent Technologies, CA, USA). After meeting expectations, qRT-PCR was performed to accurately quantify the effective concentration of the library (effective library concentration higher than 2 nM) to ensure the quality of the library. After passing the library test, Illumina NovaSeq 6000 (Illumina, USA) sequencing was performed at Novogene Bioinformatics Technology Co., Ltd., Beijing.

Quality control has been analyzed as following: Raw data were processed by in-house perl scripts, and clean data were obtained by removing reads containing adapter, reads containing ploy-N and low quality reads from raw data. Also, Q20, Q30, GC-content and sequence duplication level of the clean data were calculated. All the downstream analyses were based on clean data with high quality.

The left files (read1 files) from all libraries/samples were pooled into one big left.fq file, and right files (read2 files) into one big right.fq file. Transcriptome assembly was accomplished based on the left.fq and right.fq using Trinity (Grabherr et al., 2011 Grabherr M G, Haas B J, Yassour M, et al. (2011). Full-length transcriptome assembly from RNA-Seq data without a reference genome. Nature Biotechnology 29, 644–652. (Trinity)) with min_kmer_cov set to 2 by default and all other parameters set default.

### Differential expression analysis and functional annotation

The resulting reads were the input data for differentially expressed genes (DEGs). For samples with biological duplicates, we used DESeq2,^44^ and the object class used by the DESeq2 package to store the read counts and the intermediate estimated quantities during statistical analysis is the DESeqDataSet, with differential gene screening criteria of |log2(FoldChange)| > 1 & padj < 0.05.

Gene function annotations were selected from the following authoritative databases: protein sequence database (Swiss-Prot), Gene Ontology (GO), Clusters of Orthologous Groups of proteins (KOG/COG), Protein family (Pfam), NCBI non-redundant nucleotide sequences (Nt), KEGG Ortholog database (KO) and NCBI non-redundant protein sequences (Nr).

### KEGG and GO enrichment analysis

The GO enrichment analysis method was followed by Young et al. (2010), which was based on Wallenius non-central hyper-geometric distribution. It provided a more accurate calculation of the probability of GO term enrichment by differential genes, with GO enrichment being considered significant with padj less than 0.05. KOBAS software was used to perform KEGG functional enrichment analysis on the differential gene set.^45^ Pathway significant enrichment analysis was performed using KEGG Pathway as the unit, Hypergeometric test was applied to find out the pathway where the differential gene was significantly enriched relative to all annotated genes. KEGG pathway enrichment was likewise used with padj less than 0.05 as the threshold for significant enrichment.

### Data screening and analysis

Data from the results of growth studies, six independent parallel experiments were taken. And for gene expression study, least three independent parallel experiments were taken, organized, and calculated using Microsoft Excel 2010 and then analyzed using SPSS software (IBM SPSS Statistics, Version 20), p-values were calculated using independent samples t-test, p < .05 indicates significant, p < .01 indicates highly significant, and finally Plotting was performed using the software ORIGIN 2018 (asterisks indicate significant differences: *P < .05, **P < .01).

## Supplementary Material

Supplemental Material

## Data Availability

All relevant data are within the manuscript and its Supporting Information files.
