# Supplementary material for: Evidence for the role of sound on the growth and signal response in duckweed
Source: Plant Signal Behav. 2023 Jan 12;18(1):2163346. doi: 10.1080/15592324.2022.2163346 (PMC9839374; doi:10.1080/15592324.2022.2163346)
Supplement: Supplemental Material [file KPSB_A_2163346_SM8167.doc]

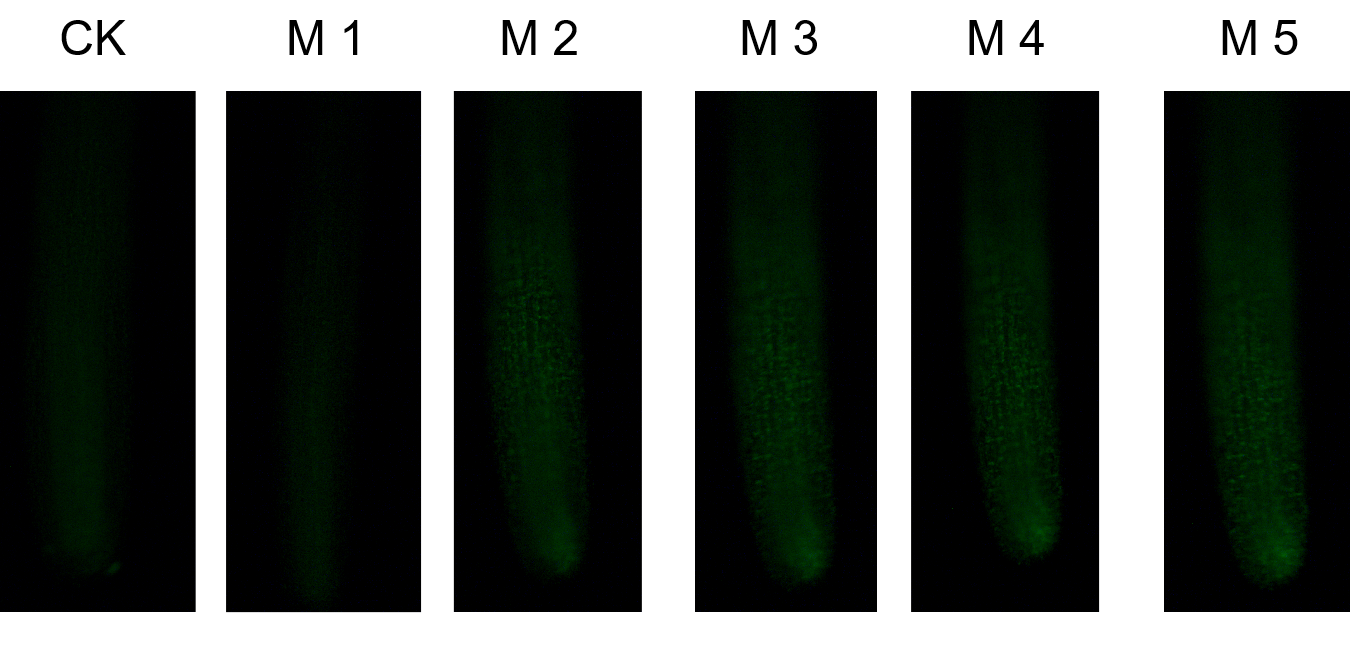


Fig. S2 The fluorescence in the transgenic duckweed subjected to different kinds of music for 30 min. CK, the transgenic duckweed treated without music; M 1, the transgenic duckweed treated with《The First Snowflakes》；M 2, the transgenic duckweed treated with《Castle in the Sky》；M 3, the transgenic duckweed treated with《Booyah》；M 4, the transgenic duckweed treated with《Let's us crunk》；M 5, the transgenic duckweed treated with《Suite for Cello Solo No.1 in G,BWV.
